# A Review of Key Technologies and Trends in the Development of Integrated Heating and Power Systems in Agriculture

**DOI:** 10.3390/e23020260

**Published:** 2021-02-23

**Authors:** Xueqian Fu, Yazhong Zhou, Feifei Yang, Lingxi Ma, Hai Long, Yujie Zhong, Peng Ni

**Affiliations:** College of Information and Electrical Engineering, China Agricultural University, Beijing 100083, China; zhouyazhong@cau.edu.cn (Y.Z.); yangfeifei@cau.edu.cn (F.Y.); malingxi@cau.edu.cn (L.M.); 2019308010225@cau.edu.cn (H.L.); 2018308010113@cau.edu.cn (Y.Z.); nipeng@cau.edu.cn (P.N.)

**Keywords:** agricultural energy, photovoltaic, bioenergy, micro energy grid, internet of things, energy internet

## Abstract

Petroleum agriculture, characterized by mechanization and chemistry, is developing rapidly in China. However, petroleum agriculture has not only brought food safety problems, but also caused great obstacles to the sustainable development of society. In view of the disadvantages of oil agriculture, we provide an upgrading plan for energy systems in agriculture. This work can help reduce carbon emissions and improve food security. We introduce the most advanced technologies in Chinese agricultural development and the technical scope includes new agricultural energy power generation, agricultural energy use and the safe operation of agricultural energy systems. We describe the detailed data of agricultural bioenvironmental and energy engineering to clarify the level of agricultural energy efficiency in China. The overall conclusion of this paper is that the deep integration of agriculture and energy internet has become the development trend of agricultural energy systems.

## 1. Introduction

Agricultural energy system is a typical integrated cooling, heating and power system. The air temperature, soil moisture, light intensity and other facility agricultural environment elements are maintained by integrated cooling, heating and power systems. Carbon dioxide from energy production can feed crops and this carbon cycle is called carbon rich agriculture. Compared with residential and building energy systems, the integration of cold, heat, electricity and gas in agriculture is more obvious. The agricultural energy system is an important scenario for the application of an integrated cooling, heating and power system (ICHPS). ICHPS can be used to adjust the thermal environment of facility agriculture and electricity systems can adjust the water and light environment of facility agriculture. Currently, agricultural production consumes 70% of the freshwater resources and 30% of the available energy on Earth [1,2]. Around the world, Israel, the United States and the Netherlands are in the leading positions in the field of agricultural energy, while China is in the development stages. Agricultural water-saving irrigation technology in Israel, biomass power generation in the United States and greenhouse horticulture technology in the Netherlands accelerate the development of agricultural energy systems. Some studies predict that by 2025, the agricultural energy consumption in China will reach 1616.1 million tons of standard coal, approximately twice the value recorded in 2016 [3]. The proportion of energy consumed by various agricultural activities also differs in different regions. Agricultural mechanical equipment is the main source of agricultural energy consumption in China, whereas fertilizer application accounts for the largest proportion of agricultural energy consumption in Iran [4], Pakistan [5] and India (approximately 50%) [6]. The energy consumed by product processing accounts for 51.14% of the total energy consumption in Brazil; agricultural mechanical equipment, fertilizer and diesel have the greatest contributions to the total energy consumption in Turkey [7]; and agricultural machinery and their maintenance consume larger amounts of energy in developed countries such as the United States and the United Kingdom [8]. With respect to developed countries, such as the United States and the Netherlands, agricultural machinery accounts for the largest proportion of energy consumption due to the high level of domestic mechanization. With respect to developing countries, such as Pakistan and India, fertilizer application and other sources of energy consumption account for the largest proportion. With respect to China, the energy consumed by agricultural machinery accounts for the highest proportion due to the vigorous introduction of advanced technologies from developed countries.

With prominent problems such as population growth, resource shortages and environmental change, sustainable development has become the focus of countries all over the world and agricultural sustainability has also attracted increasing attention [9]. By analyzing agricultural energy consumption, the existing schemes are able to evaluate agricultural sustainability [10], which will improve our understanding of the sustainability of agricultural development. Various agricultural activities, such as agricultural production, harvesting, processing, storage and sales, are accompanied by energy consumption, which substantially increases the consumption of energy in the form of fossil fuels [11,12] and carbon dioxide emissions [13]. Although an increase in agricultural energy consumption may improve agricultural production and exerts a positive effect on agriculture, the process of agricultural energy consumption also exerts a negative effect on social development and the natural environment [14]. In recent years, the problems caused by agricultural energy consumption have received increasing attention [15] and countries are also trying to reduce the negative effects of agricultural energy consumption by reducing greenhouse gases (GHG, which contains carbon dioxide, methane and nitrous oxide) emissions [16]. Most studies examining the effects of agricultural energy consumption analyze agricultural energy consumption from the perspectives of the environment and economy.

From the perspective of the environment, the rapid increase in energy consumption following development in all areas of life has led to an increase in GHG emissions, producing a series of environmental problems, such as the greenhouse effect, groundwater recession, biodiversity loss, soil pollution and water pollution [17]. The negative effect of energy consumption on the ecological environment is becoming increasingly serious [18]. Rodriguez et al. [19] investigated six environmental evaluation models and found that the ecological model was the most suitable approach for an agricultural environmental assessment, because it helped assess the environmental impact of agricultural energy consumption. Activities related to agricultural energy consumption, such as crop planting, livestock breeding and aquatic product fishing [20,21], all emit carbon dioxide and thus agricultural energy consumption is also positively correlated with carbon dioxide emissions. Using the logarithmic mean Divisia index (LMDI) to analyze the change in agricultural carbon dioxide emissions in China [22], provinces in eastern China were shown to have a developed economy and advanced agricultural technology, while the central and western provinces were behind in terms of agricultural technology. Therefore, the central and western provinces of China generally lag behind the eastern provinces in terms of agricultural energy consumption and carbon dioxide emissions [23]. Due to the excessive use of modern agricultural technology and fertilizer in China and the low fertilizer utilization rate, the environmental pollution in China is higher than in ordinary developing countries and much higher than in developed countries such as the United States.

From the perspective of the economy, an analysis of the energy costs of the four agricultural subsections in China revealed that planting and forestry have a positive impact on peasants’ income, while animal husbandry and fishery have a negative impact on peasants’ income [24]. By analyzing agricultural energy costs worldwide, irrigation water and fertilizer input account for a large proportion of the total agricultural energy costs in various countries [25]. Some scholars have recently established models to predict energy costs of agricultural machinery [26], but the cost predicting other agricultural activities must be improved. If a cost forecasting model can be established for each stage of agricultural activities to achieve an accurate cost prediction, it will help to adjust the proportion of agricultural energy input to the least energy input, obtain the greatest economic benefit and reduce agricultural energy costs. According to the current status of China, data from the National Bureau of Statistics showed that the proportions of agricultural energy consumption in 2015, 2016 and 2017 were approximately 1.915%, 1.96% and 1.99%, respectively [27]. The proportion of agricultural energy consumption continues to increase in China and the agricultural energy input is also increasing. With the increase in renewable energy production, many studies have proposed to reduce the investment of non-renewable energy in agriculture by incorporating mature renewable energy power generation technologies, such as solar and wind power generation [28,29,30,31]. Partial replacement of fossil fuels with renewable energy [32] will not only produce substantial economic benefits but also help reduce GHG [33] and carbon emissions [34]. Econometric techniques were used to analyze agricultural data from the top 12 EU countries [35] and Iran [36] and agricultural energy consumption generally had a negative impact on agricultural economic benefits. Compared with developed countries, a large part of China is still in a traditional agricultural stage and thus the agricultural economic benefits lag far behind the developed countries.

From the perspective of energy, the key problems in the development of modern agriculture in China are listed below: (1) The first problem is environment-energy-food collaborative security. The industry barrier between energy and agriculture is one of the bottleneck problems hindering the environment–energy–food collaborative security. (2) Another problem is the insufficient driving force for agricultural production. Insufficient investments in fixed assets and high energy costs are bottleneck problems hindering the development of large-scale agriculture. (3) Finally, intensification and large-scale agricultural production are problems. The low-level informatization and automation is one of the bottleneck problems hindering the development of agricultural clusters.

Currently, China is developing agricultural energy internet technology to solve these problems: (1) Key technologies for deep coupling optimization of heterogeneous energy and agricultural production, such as concentration photovoltaic and diffraction interferometry, agricultural textile power generation technology, etc., are being developed. These technologies will break barriers between the existing energy and agricultural systems, achieve the synergy of agriculture and energy, promote the development of clean energy and reduce environmental pollution. (2) The engineering mode of the integration of new energy and modern agriculture, such as agriculture–solar hybrid, fishery–solar hybrid, etc., has been proposed to break the barriers between the existing energy and agricultural industries, achieve the cross-border integration of “internet + energy + agriculture”, promote the large-scale operation of agricultural production with a new industrial mode, stimulate agricultural production investments and promote the development of agricultural scale and economy. (3) The Internet of Things (IoT) and artificial intelligence technology (AIT) have been introduced to promote intelligent agricultural production, gradually achieve the integration of IoT in agriculture and power systems, stimulate the development of agricultural production in terms of scale, cluster and intelligence and promote the transformation and upgrading of agricultural clusters.

The novel contributions of this paper can be summarized as follows. (1) The deep integration of energy and agriculture has become an important development trend and an agricultural energy system is an interdisciplinary scientific direction. For the first time, this paper systematically analyses the research status of agricultural energy systems at home and abroad. (2) This paper introduces the core technologies of agricultural energy systems from the author’s unit (i.e., China Agricultural University) and other institutions in China. These technologies will greatly promote China’s agricultural modernization and low-carbon energy production. (3) This paper introduces the agricultural energy system projects with detailed and actual data, which has a certain reference value for the construction of new projects. (4) A direction of upgrading the existing agricultural energy system is given and it should be an agricultural energy internet, which can improve the degree of coupling coordination among water, electricity, gas, heat and cold.

## 2. Research Status at Home and Abroad

This section discusses the research status of agricultural energy at home and abroad in terms of new energy power generation and energy consumption in agricultural production. In terms of agricultural energy power generation, wind power, solar energy, biomass energy and other forms of energy are mainly considered. The status of energy consumption in agricultural production is mainly analyzed from the three perspectives of planting, animal husbandry and fishery. Each subsection of this section first introduces the research status abroad and then introduces the research status in China.

### 2.1. Agricultural Energy

#### 2.1.1. Wind Power for Agriculture

Foreign scholars actively conducted projects that combine crop production and wind power generation [36,37]. Due to the limitations of wind power in agriculture [25], some studies tried to combine it with other renewable energy sources to meet demand [38,39]. The research status of wind power for agriculture in China is introduced as follows. According to Feng et al. [34], improvements in the utilization rate of agricultural land increase the wind energy potential of China by approximately 36.8%. The most abundant wind power is located in Northwest China and the population is concentrated in the southeast, but abundant agricultural land is available in the east that could be used. The development of agricultural wind power in China is valuable and feasible. Through simulation, optimization, technical and economic analyses, the most economical and reliable configuration scheme of an off-grid hybrid power system in remote rural areas was as follows: 104 kW photovoltaic modules, three 10 kW wind turbines, a 50 kW biogas fuel diesel generator (BDG), a 331 kW battery and a 99 kW converter. The annual energy production was approximately 322 MWh, which made the village independent of the main power grid and supplied power to users at a reasonable cost of $0.201/kWh [35]. The combination of photovoltaic and wind power generation is suitable for agricultural development in off-grid areas of China. In summary, China does not currently maximally exploit wind power in agriculture, with a small installed capacity and low technical level, which has great potential for development. Some practices in the United States and other developed countries in the development of wind power, such as wind power agriculture, power netlist, etc., are at the leading level of the world and China should learn these techniques.

#### 2.1.2. Solar Energy for Agriculture

The application of solar energy in agriculture is a research hotspot and one of the important ways to promote low-carbon agricultural production. At present, foreign scholars carried out a series of research on the problems and advantages of the application of solar greenhouse [40], solar air heater [41] and greenhouse coverage materials [42,43]. The research status of photovoltaic agriculture in China is initially introduced. Li et al. [44] mainly studied the social impact of a photovoltaic greenhouse. The survey showed that photovoltaic greenhouse would provide jobs and increase taxes. Most photovoltaic agriculture was designed to place photovoltaic panels on farmland or greenhouses. When simultaneously considering photovoltaic power generation and agricultural production, the more important factor was that they would be promoted without affecting the other process, rather than simply combining them. Many studies have investigated methods to improve power generation efficiency without affecting crop growth. Domestic scholars have used the principle of light for technological transformation. Wu et al. [45] designed a photovoltaic greenhouse system using biconvex Fresnel lens for photovoltaic power generation and storing the residual heat of solar energy in water. The photovoltaic cells of this system were not placed on the top of the greenhouse, no shading problem occurred and the Fresnel lens did not absorb the scattered light from the sun; thus, the scattered light was still able to be used for plant growth. In addition, some scholars considered not installing photovoltaic panels on farmland or greenhouses, but installed floating photovoltaic panels on the water, namely, floating photovoltaic panels. Zhou et al. [46] used floating photovoltaic power and hydropower generation in a complementary operation and considered the maximization of the ratio of water storage to storage capacity and water supply to water demand to improve the water-food-energy synergy. In some areas of China, the high sunlight intensity at noon produces an excessively high temperature in the greenhouse. From the perspective of reducing the temperature in the greenhouse while generating electricity, the solar shading by photovoltaic panels is an advantage that may be exploited in some areas with a high sunlight intensity. Feng et al. [47] developed a new type of solid composite parabolic concentrator as a transparent covering material for greenhouses that reduces the light transmittance at noon and the unobstructed light is used for photovoltaic power generation. Numerous agricultural photovoltaic power generation industries market their own products. China has applied photovoltaic power generation to many aspects of agricultural production. Xue et al. [48] described the use of photovoltaic power generation for wastewater purification and pumping agricultural water; a mode of “power generation and aquaculture” using the agricultural environments such as greenhouses and fishponds, was also shown to generate electricity, plant and breed at the same time. Liu et al. [49] matched photovoltaic power generation with movable sprinkler irrigation equipment (SMSIE), which saved both water and energy. Li et al. [50] used a solar water pumping system to simultaneously solve the energy crisis and water crisis and increased the crop yield in developing countries far away from the grid. Studies have also examined the combination of photovoltaic power generation and solar heat collection for bee breeding in China. He et al. [51] proposed a new type of solar beehive system. The system used the solar collector to heat beehives and solar photovoltaic power generation to drive a fan to cool beehives, ensuring that the beehives would maintain a better thermal environment and thus improve the yield and quality of honey. Photovoltaic power generation not only directly provides electric energy but also electrolyzes water to produce hydrogen with the generated electricity. Anifantis et al. [52] proposed a system that used the generated electricity to electrolyze water and produce hydrogen during the day and then converted hydrogen into electricity to provide energy for a ground source heat pump (GSHP) using fuel cells at night, thereby heating the greenhouse. The disadvantage was that the system was substantially affected by climate and the efficiency was only 11%. Photovoltaic agriculture involves both power generation and heat storage. Cao et al. [53] mentioned the use of photovoltaic heat storage to provide heat energy for a greenhouse in winter or at night to prevent crop frostbite. Photovoltaic agriculture in China is often combined with irrigation and pump water for several reasons. First, China is a large agricultural country, but it is also a country with a water shortage. The amount of water consumed by agriculture, which requires a large amount water resources, can be reduced by improving the utilization rate of irrigation water. Second, irrigation and pump water are closely related to agriculture and photovoltaics are closely related to agriculture because of the requirement for land. The combination of photovoltaics, irrigation and pump water adapts to the trend of combining agriculture and energy. Third, photovoltaic power generation is mostly suitable for areas far away from the grid, such as arid areas in Northwest China, which often have a limited water supply. The combination of photovoltaic, pumping and irrigation systems will not only solve the problem of power generation in these areas but also reduce the waste of water resources to achieve an optimal situation. Fourth, photovoltaic power generation is characterized by its intermittent nature, which exerts a very adverse effect on the stability of the power grid, while the irrigation and pumping system can be used as buffer to improve the stability of power grid. In the long term, the development of photovoltaic agriculture is very important for the agricultural transformation of China; in the short term, photovoltaic agriculture is an effective measure to solve the current plight of the photovoltaic industry in China to some extent.

#### 2.1.3. Biomass Energy for Agriculture

The demand for crop residues should not affect food production due to the trends in bioenergy and crops should be planted for food production rather than energy [54]. The research status of agricultural biomass energy in China is initially introduced. Amaducci et al. [55] comprehensively evaluated the biomass energy potential of China. In 2016, the total potential of biomass energy resources in China reached 32.69 EJ, which is equivalent to 27.6% of the domestic energy consumption and the development of biomass energy substantially reduced emissions in China. The available biomass energy of Heilongjiang Province doubled from 2003 to 2013 and it was very important to strengthen the regional practice of water resource management and irrigation in agricultural systems for the effective utilization of agricultural biomass resources [56]. This study confirmed the potential of developing biomass energy in China. Liu et al. [57] discussed the relevant policies on biomass energy and the future trends in biomass energy in China. Guan et al. [58] analyzed the current status and industrial policy of biomass briquette in China and discussed the problems and challenges in the standard system, legislation, development plan, incentive policy and other aspects of the biomass briquette industry. This study was related to the development of a policy for biomass energy regulation. Sun et al. [59] established the mechanism for supplying crop residues and the value-added process model. Monte Carlo and risk tolerance methods were used to explore the interactions among the transportation cost rate, unit cost of crop residues, basic price of crop residue sales and coal price, which provided a reference for feasibility planning and policy making related to biomass energy projects in China. Wang et al. [60] proposed that the direct economic cost of straw power generation was 0.45 yuan/kwh, of which 89% was attributed to fuel purchase and transportation costs. Compared with coal power generation, its economic competitiveness was at a disadvantage, but coupled with external costs, it was at an advantage. The price of biomass waste was the most sensitive factor affecting the economic benefits of biomass power generation. This paper mainly expounded on the development of biomass energy in China from the perspective of the economy. Wei et al. [61] summarized five stages of agricultural waste energy utilization in China and indicated that agricultural waste utilization in China was changing from planting wastes to breeding wastes.

Many studies have examined agricultural biomass energy technologies related to biomass gasification in China that produces hydrogen, methane, ethanol and other clean gases and strives to improve the gas production rate. Dai et al. [62] studied the effects of straw ingredients and surface properties on biogas production. The methane production potential of typical straw ranked as follows: corn > wheat > sweet sorghum > rice straw. The mineral composition of straw was closely related to methane production and silicon inhibited the production of methane from straw. These results provided theoretical support for improvements in the gas generation rate of agricultural wastes. Hydrogen and methane were recovered in a two-stage biological process using the waste residue from bioethanol fermentation as materials. The COD removal rate reached up to 81% and 0.3% H2 and 72.8% CH4 were converted. The process improved the energy utilization rate through secondary utilization and produced more clean gas [63]. The feasibility of a low temperature alkali/urea pre-treatment of rice straw to improve the hydrogen production rate was studied. After the pre-treatment, the maximum hydrogen production was 22.08 mmol/L and the energy transformation ratio was 9.76%. Compared with the control group without the pre-treatment, these parameters were increased by 161.92% and 56.91%, respectively [64]. Most studies of biomass technology in China have focused on biomass gasification technology, while biomass briquettes and other related technologies that utilize biomass energy have rarely been investigated, which is also a limitation of the development of agricultural biomass energy in China.

#### 2.1.4. Status Analysis

Agricultural biomass energy, wind energy and solar energy are becoming the “engine” of global energy transformation. Bio-based materials are the only alternative to non-renewable chemical materials and have become the predominant direction of biomass conversion and important sources of chemical materials. Biomass materials have become one of the main goals of global agricultural production. The modern agriculture, using the complementary approach of wind and solar energy, will enable land resources to be shared and promote the integrated development of new energy technology and modern agriculture.

### 2.2. Energy Use in Agricultural Production

#### 2.2.1. Planting Industry

Foreign planting industry mainly manages agricultural machinery [65,66,67], photovoltaic greenhouses [68,69,70,71,72], irrigation [73,74,75,76,77,78,79] to save energy and reducing emissions [80,81,82]. In the analysis of planting agriculture in China, the logarithmic mean divisia index (LMDI) method and data envelopment analysis (DEA) model are widely used to study the effect of energy consumption on environmental factors. Zhen et al. [83] decomposed agricultural driving forces into cultivated land and labor driving forces using the LMDI method and found that agricultural energy consumption was associated with a significant increase in domestic GHG emissions. DEA was used in recent studies to evaluate the efficiency of agricultural production. Since the results of the evaluation are easily affected by environmental variables [84], Zheng et al. [85] designed a more reliable evaluation system for a more accurate estimation of agricultural production efficiency and to determine the energy input of the agricultural system and reduce energy loss. Aiming at addressing the problem of the low energy utilization of domestic agricultural machinery, a multi-objective particle swarm optimization algorithm was used to replace the original single-objective algorithm, which might effectively improve the output power of the Stirling engine [86]. For photovoltaic greenhouses, multi-energy complementary control and modern agricultural farming technology were combined to achieve the local consumption of photovoltaic greenhouse power generation [87]. By establishing the optimal energy operation model, the local consumption of photovoltaic greenhouse power generation was maximized, which was conducive to alleviating photovoltaic poverty in western poverty-stricken areas. Regarding the use of fertilizers, the optimal nitrogen ratio (ONR) of the in-season nitrogen management strategy (INM) was used to maximize crop yields, balance the supply and demand of nitrogen fertilizer and reduce the negative effect of nitrogen fertilizer use on the environment [88]. Regarding irrigation water, Zhang et al. [89] compared Bowen’s ratio method with the soil water balance method and found that Bowen’s ratio method more accurately estimated the evapotranspiration of vineyards in arid areas, which was conducive to the detection of evapotranspiration in vineyards and the reasonable control of water resource input to vineyards. An intelligent water-saving irrigation system based on a wireless sensor network was designed and achieved a certain water-saving effect [90]. The proportion of effective irrigation in agriculture in China increased from 44% in 1993 to 57% in 2012 [91], which also confirmed efforts designed to save agricultural irrigation water in China.

Compared with the development of the foreign planting industry, the planting industry in China exerts a certain effect on the optimization of energy consumption activities, such as agricultural machinery, photovoltaic greenhouses, fertilizer use and irrigation energy consumption, but some problems persist, such as an insufficient level of automation of agricultural machinery and insignificant water-saving effect of intelligent water-saving irrigation system. Additionally, key technologies for predicting and actually measuring agricultural fuel consumption are still lacking.

#### 2.2.2. Animal Husbandry

Foreign animal husbandry mostly uses artificial intelligence algorithm [92,93,94,95,96] to predict its fossil fuel and power consumption, combined with life cycle assessment (LCA) [97] and other methods to study its impact on the environment [98] and used modern equipment such as ground water heat pump (GWHP) system [99] to reduce heating energy consumption. With the aim of conserving energy and reducing emissions from animal husbandry in China, Islam et al. [100] conducted a study at a hoggery in Beijing and found that the combination of a ground water heat pump (GWHP) system and irrigation system conserved energy during heating while effectively avoiding pollution caused by PM2.5. Based on the energy balance equation (EBE), the dynamic heat exchange model of the pigsty was established [101] and the performance of EBE model was evaluated using an adaptive network-based fuzzy inference system (ANFIS). The model was able to save 358.301 kWh of power consumed by the pigsty in 87 days. The “pig-biogas-fish” ecosystem in Jingzhou City, Hubei Province, China was analyzed using a life cycle assessment (LCA) method. The non-renewable energy consumption intensity of the system was lower than the traditional animal husbandry system and it displayed a better renewability and greenhouse gas emission reduction capacity [102]. Ventilation of a poultry house is also one of the energy-consuming activities of animal husbandry. The energy consumption of broiler cooling and ventilation may reach 39.5% of the total power consumption. Therefore, Du et al. [103] proposed a combination of the ventilation energy recovery system of poultry houses with small wind turbines. After applying the combined system in Chengdu City, Sichuan Province, China, the system recovered approximately 13.5% of the ventilation energy and generated approximately 2074 kWh of energy per year [104]. Due to the large amount of electric energy, steam and hot water used in dairy processing, Wang et al. [105] proposed a solar cogeneration system based on parabolic trough hybrid photovoltaic (PVT) collector for use in dairy farms. The system provides 14% electric energy, 52% steam and 40% hot water for dairy processing.

Compared with the status of development of animal husbandry at home and abroad, animal husbandry in China is in a critical period of transformation from traditional to modern practices and its development is currently undergoing a rapid growth stage. The modernization, standardization and scale of animal husbandry in China are in the stage of rapid improvement, but due to regional differences, the modernization, standardization and scale of animal husbandry are not sufficient. Therefore, compared with Western countries, animal husbandry in China is still plagued by problems such as high energy consumption and environmental pollution.

#### 2.2.3. Fishery

In foreign countries, the fishing industry relies heavily on fossil fuels. A series of experimental research was carried out on energy-saving research on fishing vessels [106,107], fishing gear [108,109,110], fishing ports [111,112,113,114,115] and other sources of fishery energy consumption. For the recirculating aquaculture systems (RAS) in China [116], which are a cooperative combination of fish culture (aquaculture) and nutrient solution for soilless plant production (hydroponics), vegetables and fish in RAS grow rapidly at higher temperatures [117] and thus, the system must be heated. Le et al. [118] proposed a combination of a spiral coil heat exchanger and thermal energy storage (TES) unit and found that it reduced energy consumption to a greater extent than the electric heater and submersible heater [119]. The electrochemical method was used to treat wastewater from a prawn culture pond on the east coast of Zhoushan [120]. Due to the excellent conductivity related to the high salinity of seawater, the energy consumed by this method was lower than the traditional wastewater treatment method. In addition, a new type of microalgae-bacterial biological fuel cell (MBBFC) was designed for aquaculture wastewater treatment and energy recovery [121]. Since fishery refrigeration consumes more energy in fishery activities, Yuan et al. [122] proposed the use of a solar-assisted hybrid power generation and refrigeration cycle system based on ocean thermal energy conversion (OTEC) to provide power for fishery refrigeration and reduce the power pressure related to refrigeration. Using a self-powered electrochemical water treatment system with a triboelectric nanogenerator (TENG) combined with seawater electrolysis technology, the energy consumption and costs of algae removal and sterilization in fishery were effectively reduced [123].

In an analysis of foreign and Chinese fisheries, although China is the world’s leader in freshwater aquatic species breeding technology and marine fishing technology, current research on the energy conservation of fisheries in China has not been performed at a sufficient depth and energy-saving research on fishing vessels, fishing gear, fishing ports and other sources of fishery energy consumption is still lacking.

#### 2.2.4. Summary of the Current Status

Generally, China is a large agricultural country, but the high input and low recovery in its agricultural development are persistent problems. The competitiveness of agricultural development in the world is not high. The economic and technical investments of energy-saving control measures related to planting industry are higher and more in-depth research has also been conducted to meet the domestic food demand. However, the investments in animal husbandry and fishery are lower. As a result, the development of domestic animal husbandry and fishery technology lags behind Sweden, Norway and other Western countries.

## 3. Key Technology

This section introduces the status of research into key technologies for new agricultural energy power generation, agricultural energy use and the safe operation of agricultural energy systems. Regarding new agricultural energy power generation, the agricultural textile power generation technology, concentrated photovoltaic and diffraction interference technology and microbial fuel cell technology are introduced, which provide new insights into the source of agricultural energy. In terms of agricultural energy use, the greenhouse heating technology, nitrogen fixation and sterilization technology based on computational fluid dynamics (CFD) and energy prediction model (EPM) are introduced. These technologies are very important for reducing agricultural energy costs, environmental protection and food safety. Finally, the technology for performing safety analyses of the agricultural micro-energy network and photovoltaic agricultural IoT technology are introduced. These two technologies play a vital role in the stable operation of agricultural energy systems and agricultural IoT. The key technologies described in Section 3.2.2 and Section 3.3.1 are proposed by the College of Information and Electrical Engineering, China Agricultural University. The related technologies to be discussed in this paper are listed in Table 1. The core principles and benefits of each technology are simply compared and analyzed and the detailed discussion will be presented later.

### 3.1. New Agricultural Energy

#### 3.1.1. Agricultural Textile Power Generation Technology

**Figure 1:**
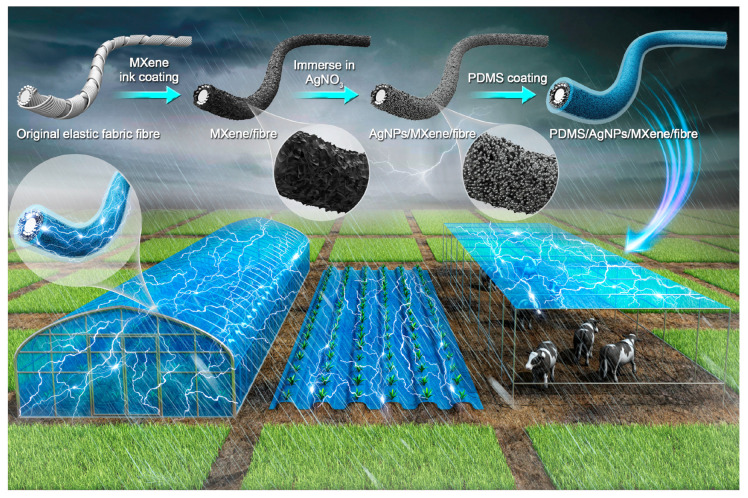
The fabrication of TENG yarn and its application in an agriculture field.

Jiang et al. [124] covered the surface of agricultural textiles with two special materials: conductive titanium carbide nanomaterials and non-conductive polydimethylsiloxane (a high molecular weight polymer). The polymer is waterproof and transfers electrons with rainwater in the environment. The titanium carbide sensor electrode not only has high conductivity, but also enables the surface polymer to capture electrons because of its high electronegativity. The intelligent agricultural textiles produced using this technology exploit the electron transfer and flow of rainwater to generate an electric current and continuously supply energy for intelligent agriculture (Figure 1). Based on the experimental data, a voltage of 7.7 V can be generated from 3 cm long yarn under a continuous force of 9.5 N. Therefore, with the aims of fabricating novel agricultural protection materials, preserving heat, shading, conserving soil and water, drainage and irrigation and the maintaining the seed cultivation functions of agricultural textiles, this technology can also continuously obtain energy from the agricultural environment to provide a driving force for smart agriculture and achieve the “passive real-time perception” of agricultural information.

#### 3.1.2. Concentrating Photovoltaic and Diffraction Interferometry Technology

**Figure 2:**
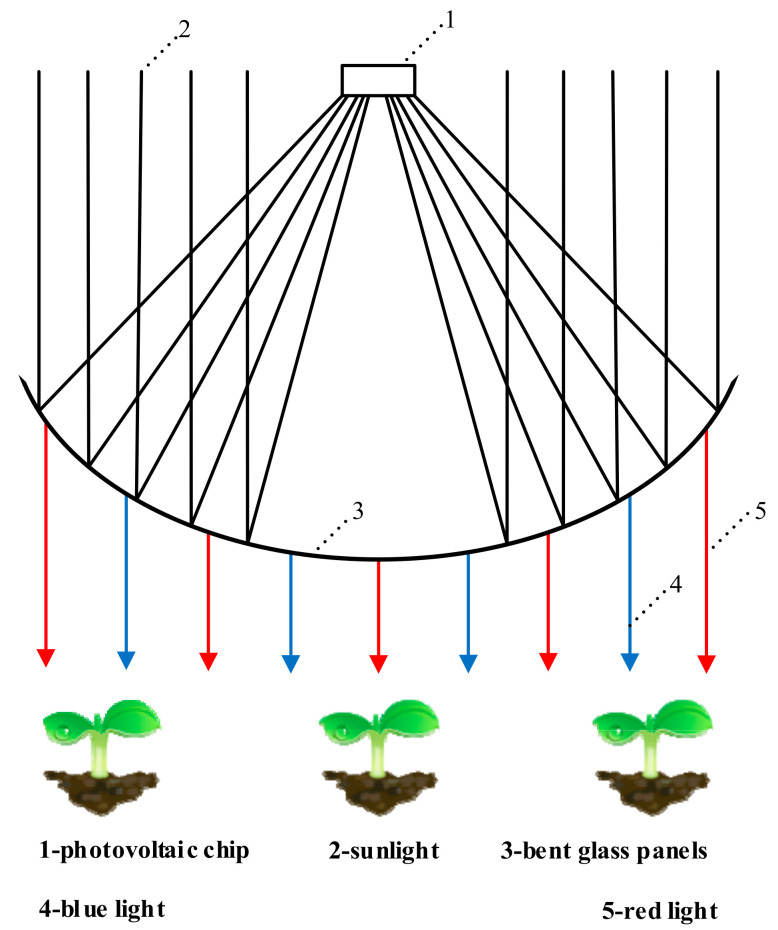
The basic principle of the novel agricultural photovoltaic system.

Liu et al. [125] combined concentrating photovoltaic technology with diffraction interferometry technology to design a new type of agricultural photovoltaic power generation system. The working principle of the system is shown in Figure 2. The curved glass panel is covered with a multi-layer dichroic interference film that transmits the red and blue light in sunlight to promote photosynthesis in crops and the remaining wavelengths of sunlight are reflected for photovoltaic power generation. The system is also equipped with dual tracking components to ensure that the reflected sunlight is always focused on the concentrating solar cells during the day and at different seasons of the year. Based on the experimental data, the average efficiency of the CPV system is 6.80%, a value that is higher than the traditional Sonneveld system (3%). The leaf surface temperature of the crops grown under the system is reduced by 2–4 °C and the water evaporation is reduced by 26%. The competition between photovoltaic power generation and crops has always been a problem in photovoltaic agriculture. The combination of concentrated photovoltaic technology and diffraction interference technology effectively establishes a balance between power generation and plant growth. In addition, this technology also reduces the evaporation of moisture from the farm, which is a crucial advantage for many countries (such as China and Israel) with scarce water resources.

#### 3.1.3. Microbial Fuel Cell Technology

**Figure 3:**
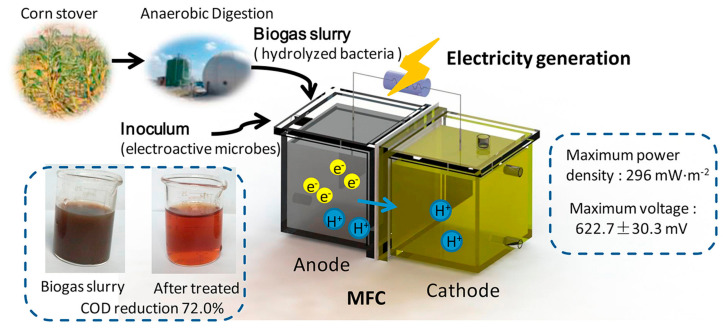
Schematic of the system.

Wang et al. [126] designed a dual-chamber microbial fuel cell with corn stalk biogas slurry as the anode (Figure 3). The anode compartment and cathode compartment of the biofuel cell are separated by a proton exchange membrane and the proton exchange membrane is pre-treated with an H_2_O_2_ solution (30%, 80 °C), deionized water and H_2_SO_4_ solution (0.5 mol/L) for 1 h and then washed three times with deionized water. The carbon felt (5 cm × 5 cm) is uniformly arranged in the anode and cathode electrode chambers and is connected by a titanium wire with a constant resistance (1000 Ω) under normal conditions. The distance between the two electrodes is 6.5 cm. The constructed microbial fuel cell is able to be quickly started and maintain a stable power generation capacity (622.7 ± 30.3 mV) for 10 days. When the external resistance is 200 Ω, the maximum power density of the fuel cell is 296 mW/m^2^. The chemical oxygen demand and the rate of ammonium nitrogen removal on the 16th day were 72.0% and 43.9%, respectively. The constructed microbial fuel cell effectively degrades the organic matter in the biogas slurry and generates electricity, representing a new method for the recycling of biogas slurry.

### 3.2. Agricultural Energy Use

#### 3.2.1. Greenhouse Heating Technology Based on Computational Fluid Dynamics (CFD) and the Energy Prediction Model (EPM)

**Figure 4:**
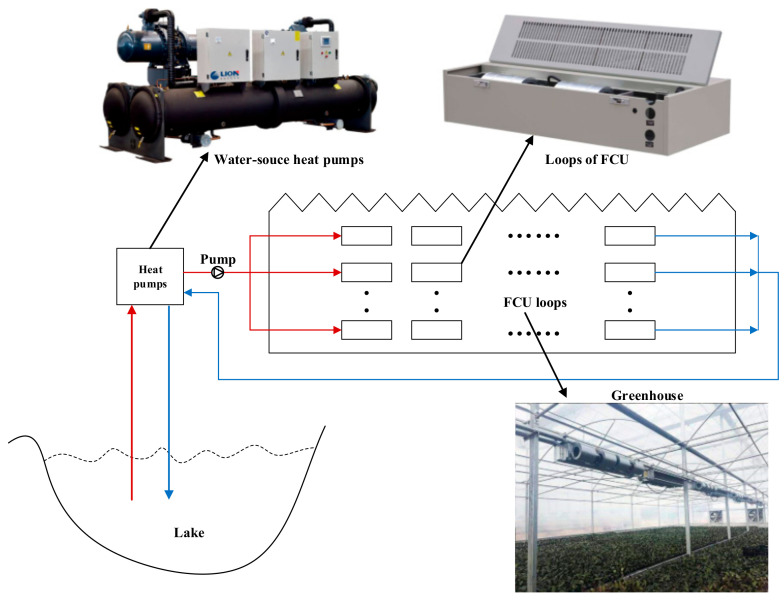
Schematic of a greenhouse with surface water source heat pump system.

Chen et al. [68] proposed a greenhouse heating control method based on computational fluid dynamics (CFD) and the energy prediction model (EPM) to reduce the energy consumed by agricultural greenhouses. First, based on the principle of low Reynolds number K-ε turbulence and using the radiative heat transfer discrete coordinate model and the plant porous medium method, a greenhouse heating CFD model considering plant sensible heat and latent heat exchange is established. Second, the thermal performance of the greenhouse and the priority order of the fan-coil unit (FCU) under different heating conditions are analyzed. According to the heating efficiency and temperature uniformity, the priority of each FCU loop is predicted and the priority database of the control system is generated. Based on the thermal balance, EPM is used for the online forecasting and optimization of the greenhouse energy demand. Combined with the priority of the FCU loop in the offline CFD simulation, a surface water source heat pump greenhouse heating control system based on CFD-EPM was developed (Figure 4). Compared with the traditional multi-zone independent control (CMIC) method, this technology has the potential to conserve approximately 8.7%–15.1% of the energy and the control temperature deviation is reduced to 0.1 to 0.6 °C.

#### 3.2.2. Modern Physical Agricultural Engineering Equipment Technology

Traditional agricultural production mainly relies on biological and chemical technologies. While producing the food supply, it also encounters some bottlenecks, such as soil and water pollution, excessive use of pesticides that result in food pesticide residues and pests and diseases occurring annually. Research conducted by the College of Information and Electrical Engineering, China Agricultural University combines high voltage, an electromagnetic field, plasma, pulse power, power electronics and other electrical technologies with agricultural production processes and explores some novel application technologies. Some methods have initially been proposed in China and abroad, providing many new solutions for agricultural weight loss and drug reduction, reduced levels of pesticide residues and increased yield.

**Figure 5:**
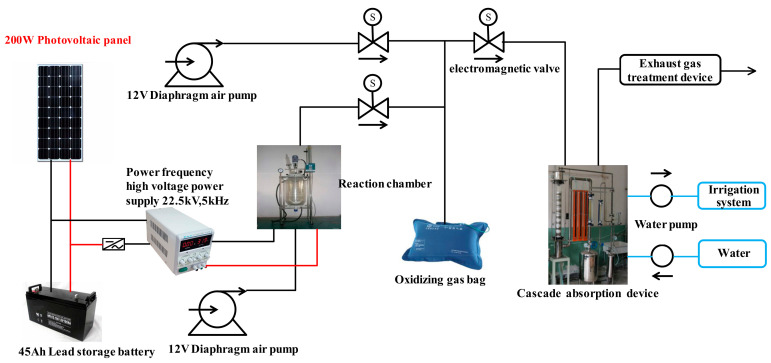
Basic structure of the nitrogen-fixing prototype.

The solar low-temperature plasma nitrogen fixation system developed by the College of Information and Electrical Engineering, China Agricultural University is shown in Figure 5. It mainly consists of four parts: a plasma discharge reaction chamber, an oxygen mixing device, a nitrogen oxide absorption device and an exhaust gas treatment device. Solar energy is the energy source, water, air and calcium carbonate-containing rocks are used as raw materials to simulate the process of natural lightning discharge that produces calcium nitrate. Gaseous nitrogen oxides are generated through plasma discharge and liquid nitrogen fertilizer is generated through a specific absorption device before it is ultimately applied in agricultural production. This approach is very important to solve the problem traditional artificial nitrogen fixation that requires fossil energy consumption and emits large amounts of carbon dioxide. Yang et al. [127] also studied the effects of different frequency power supplies on the output of NOx using this system. High-frequency power supplies had the advantages of low energy consumption, a small size, high reaction efficiency and insensitivity to gas flow. The low-frequency power supply consumes approximately three times as much energy as a high-frequency system to produce the same amount of nitrogen oxide in water.

**Figure 6:**
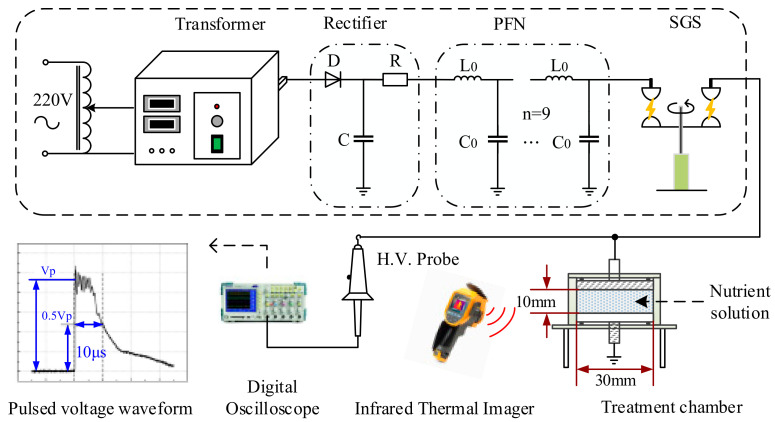
Schematic of the PEF disinfection system.

In the closed soilless culture system, the disinfection of the circulating nutrient solution is the key step to avoiding the spread of pathogenic bacteria and causing disasters. Researchers at the College of Information and Electrical Engineering, China Agricultural University have designed technology for the sterilization of the plant nutrient solution based on a high voltage pulsed electric field (PEF) (Figure 6). The implementation of this technology effectively solves the problem of slow crop growth and other issues caused by traditional sterilization methods, such as ozone and ultraviolet radiation. Zhong et al. [128] selected Fusarium oxysporum as a typical pathogen and Hoagland solution as the living environment of the pathogen to study the effect of the (PEF) intensity on the bactericidal effect. The 18 treatment compartments were randomly divided into six groups and each group was administered a different (PEF) intensity (E: kV/cm) for 10 s and the initial microbial density of each test nutrient solution was 4.15 × 105 CFU/mL. The disinfection efficiency increased from 97.2% to 99.47% as the intensity of the (PEF) increased from 4 kV/cm to 8 kV/cm.

### 3.3. Smart Agricultural Energy Systems

#### 3.3.1. Technology for Analyzing the Safety of the Agricultural Micro-Energy Network

**Figure 7:**
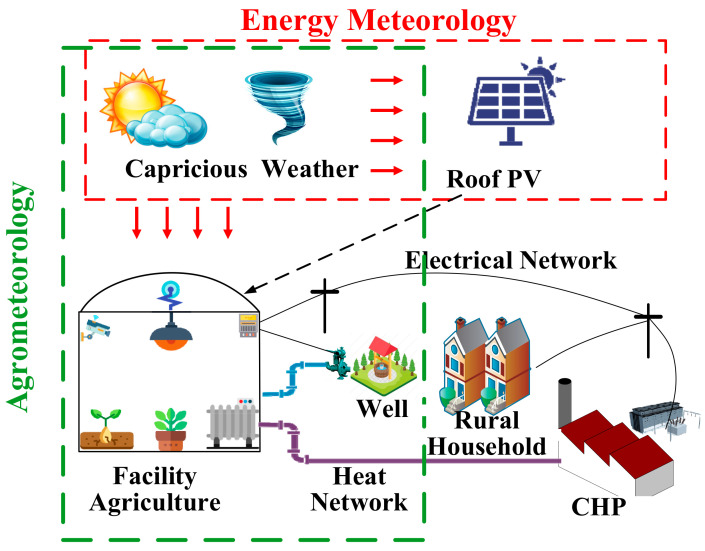
Interaction between meteorological energy and agricultural meteorology.

The micro-energy network of agricultural parks is a trend in the development of energy systems for agricultural parks, which promotes the development of facility agriculture, but it is vulnerable to meteorological influences and safety problems tend to occur under extreme weather conditions. Researchers at the College of Information and Electrical Engineering, China Agricultural University [129] have designed a method for analyzing the safety of micro-energy networks in agricultural parks, because the light environment and thermal environment of facilities suitable for cucumber growth in greenhouse require energy systems to provide electricity and heat for regulation. This method quantifies the effects of agricultural meteorology and energy meteorology and analyses the effects of weather changes, the photovoltaic panel layout and grid connection of facility agriculture on the safe operation of micro-energy networks in agricultural parks. The contribution of this article is the consideration of the interaction between meteorological energy and agricultural meteorology. As shown in Figure 7, the spatial coupling model and energy coupling model of the energy system and the agricultural system were established.

Spatial coupling model: Spatial coupling has created conflict between photovoltaic power generation and crop growth. The area and layout of photovoltaic panels affect the environmental temperature and light of facility agriculture. The demands of power generation and farming for daylight must be balanced. Equation (1) is used to calculate the effect of solar radiation on the light environment of a facility greenhouse.

(1) $$I_{\mathrm{solar}}=G\times \tau_{z}$$

where *I*_solar_, W/m^2^, is the indoor solar radiation of the PV greenhouse and *τ*_z_ is the light transmittance, with limits of 32.64% and 80.96% and an average value of 66.27%.

Energy coupling model. The following mathematical model of a Philips metal halide lamp is used:

This is example 1 of an equation:

(2) $$I_{E}=I_{\mathrm{indoor}}-I_{\mathrm{solar}}$$

(3) $$I_{E}=\frac{N\times p_{E}\times 94.5}{A\times \Phi_{0}\times C_{1}\times C_{2}}$$

where *I_E_*, lx, is the average illumination; *A*, m^2^, is the greenhouse area; Φ_0_, lm/(m^2^• lx), is the luminous flux; *C*_1_ and *C*_2_ are correction factors; *N* is the number of lamps; and *p*_E_, W, is the lamp power (energy coupling variables, greenhouse power consumption and energy network power supply); and the units of 94.5 are LM/W. For the metal halide lamp, 1 klx is equal to 14.4 μmol/(s•m^2^).

#### 3.3.2. Photovoltaic Agricultural Internet of Things (PAIoT)

**Figure 8:**
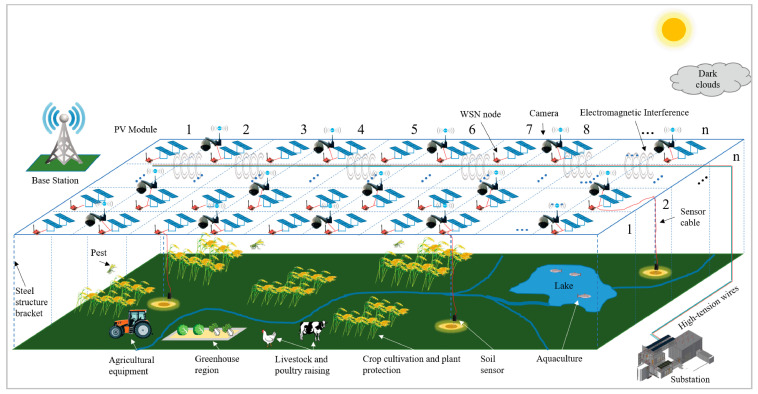
Schematic of PAIoT.

In the future, a large amount of data will need to be transmitted, including images and audio and video files. Regardless of the type of equipment and technology used, the transmission of this information will consume a large amount of energy and the energy supply will be a bottleneck for future smart agriculture. Photovoltaic Agricultural Internet of Things (PAIoT) integrated agricultural production with renewable energy power generation and control through an IoT platform [130]. PAIoT can realize the docking of supply and demand through sharing information and then photovoltaic generation can match multiple types of agricultural power demands. First, the PAIoT system combines agriculture and photovoltaics by building photovoltaic panels above the farmland to protect crops from natural disasters and provide energy for agricultural production. Second, by installing sensors and equipment on the photovoltaic panel support, field information about the production area can be collected while reducing deployment costs, such as field planting, facility gardening and livestock and poultry breeding. A stable power supply is provided for WSN nodes through photovoltaic generation (Figure 8). Finally, the system also performs data fusion and data processing to make intelligent decisions and provide early warnings for agricultural production and photovoltaic power generation panels, which will promote the intelligent development of photovoltaic power generation and agricultural production.

## 4. Demonstration Project

At present, the engineering mode of deep coupling of new energy and agricultural production is being widely developed in China. Taking photovoltaic agriculture as an example, more than 2000 GW of photovoltaic agricultural projects have been completed. They are mainly concentrated in the provinces represented by Henan and Hebei, which have large electricity demand for agricultural production and rural life, and in relatively remote areas represented by Inner Mongolia, Xinjiang, Ningxia and Qinghai, which have abundant solar energy resources [131].

**Figure 9:**
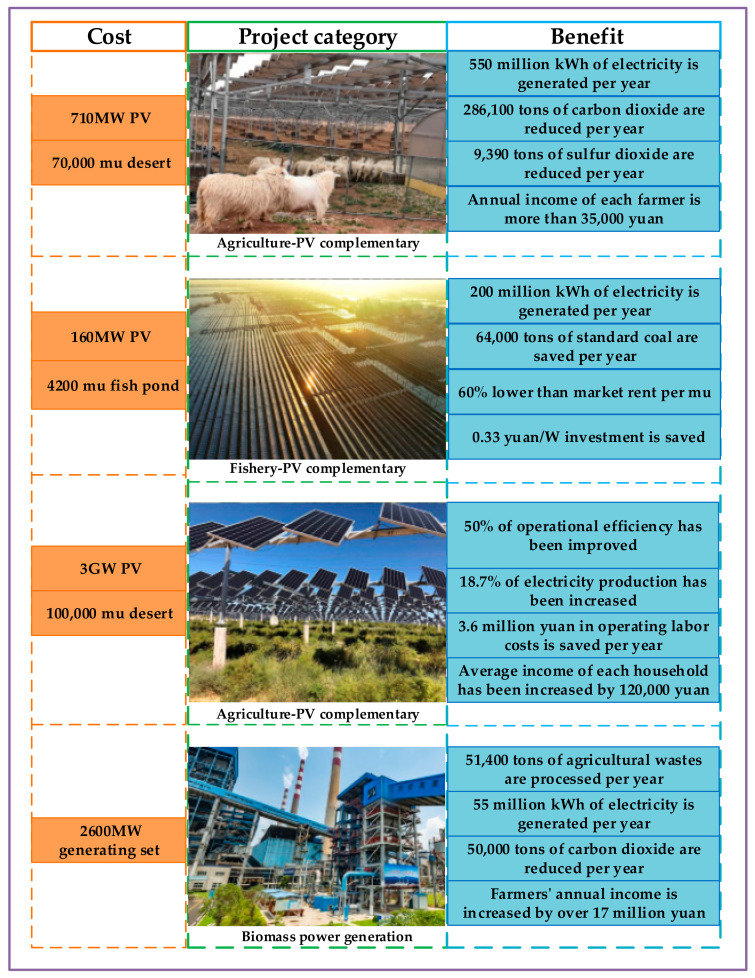
Demonstration projects of integrated agriculture and new energy systems.

Here we introduce four representative projects based on agriculture-PV complementary or fishery-PV complementary. Data on costs and benefits of each project is shown in Figure 9, where the ecological and economic value of each project is displayed. Project 1 is located in Kubuqi Desert, the seventh largest desert in China. The project adopts a five-in-one compound ecological solar sand control model of “desert control + grass planting + farming + power generation + poverty alleviation” [132,133]. Project 2 is located in Hefu Town, Nanxun District, Huzhou City, Zhejiang Province. The project combines the photovoltaic power station with the local traditional mulberry fishpond, integrating aerial power generation, underwater aquaculture and water sight-seeing [134,135]. Project 3 is located in Binhe New District, Yinchuan City, Ningxia Province. The project is responsible for the ecological control of desertified land, plants local characteristic industry wolfberry and builds a photovoltaic power generation project above the wolfberry plants [136,137]. Project 4 is a demonstration project of agriculture and forestry biomass power generation in Xiangyang City, Hubei Province. It can generate biomass ash while using agricultural and forestry wastes to generate electricity, which can be used as agricultural organic fertilizer to improve the soil environment [138,139].

The above four projects are representative engineering models of integrating agriculture and new energy in China and all of them offer both environmental and economic benefits. First, all of them use make full use of clean energy to generate electricity, such as solar energy and biomass energy, reducing the emissions of polluting gases such as carbon dioxide. Second, all of them have complementary advantages in agriculture and energy, which concludes production cost reduction and increment benefit. One can improve the benefit from agricultural land via agriculture-PV complementary and fishery-PV complementary. Biomass power generation is companied with agricultural waste utilization and agricultural organic fertilizer is companied with energy waste utilization, resulting in the cost reduction of energy and agricultural production.

## 5. Development Trends

With the increasingly urgent need to integrate new energy technology and modern agriculture, as well as the development of energy internet technology, the theory of park-level agricultural energy internet has been developed. The park-level agricultural energy internet has distinctive interdisciplinary characteristics. It aims to promote the innovative and coordinated development of modern agriculture and energy internet through interdisciplinary research. It is the material and technological foundation for the development of smart agriculture and an inevitable trend to enhance supply-side structural reform of the rural energy supply in China.

**Figure 10:**
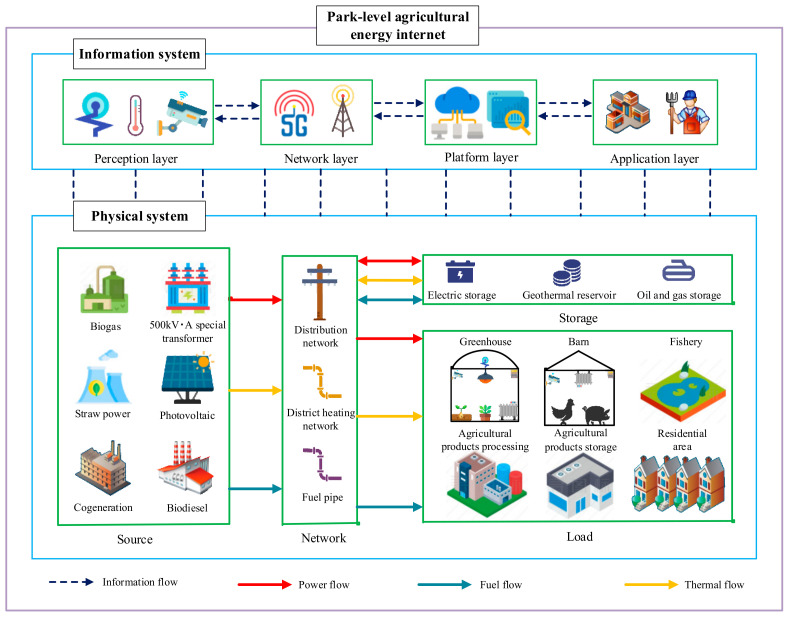
Framework diagram of the park-level agricultural energy internet.

The information system of the park-level agricultural energy internet consists of four parts: a perception layer, network layer, platform layer and application layer. The key technologies mainly include Internet of Things in power systems (IOTIPS) and agricultural Internet of Things technology (Figure 10) [140].

In the perception layer, temperature and humidity sensors, spectral sensors, current sensors, voltage sensors and smart meters are mainly used to achieve real-time perception and collect information from the four parts of the “source-network-storage-load” physical system and the field environment of agricultural production. In the network layer, communication networks, such as fiber private networks, LTE power wireless private networks and mobile internet, are mainly used to efficiently transmit data collected by the perception layer. In the platform layer, advanced technologies, such as big data, cloud computing and artificial intelligence, are mainly used to analyze and process the data transmitted from the network layer to provide a data foundation for various applications. The application layer provides convenient services for related users and departments by analyzing the data processed by the platform layer and generates various decision instructions, which are transmitted to the relevant control equipment through the communication network to control and optimize the energy flow and agricultural production environment.

**Figure 11:**
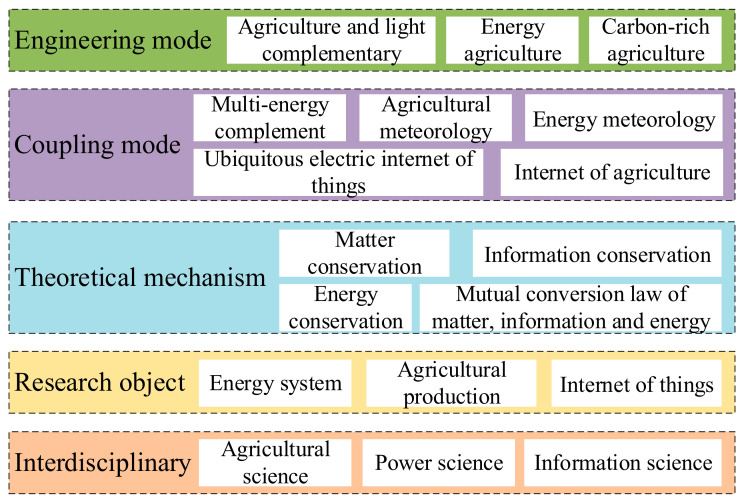
Theoretical system of the park-level agricultural energy internet.

Facility agriculture science, electric power science and information science intersect to form the theory of the park-level agricultural energy internet. The sustainable coordinated development of energy systems and agricultural production is the main line and the disturbances in agrometeorology and energy meteorology are considered. Based on the basic characteristics of material flow, energy flow and information flow, the coordination of agricultural material, energy and sensor information is achieved. The coupling of information and energy is the essential feature of the energy internet and the coupling of information, material and energy is the essential characteristic of park-level agricultural energy internet. The theoretical system of the park-level agricultural energy internet is shown in Figure 11 [141].

The value of agricultural energy internet in agricultural production includes several factors that are listed below.

Automation uses the Internet of Things to fully perceive the operation of the agricultural energy system, the combination of weather information and artificial intelligence technology to predict the output of new energy and the relevant information from the agricultural Internet of Things to form energy supply matching with farmers and make the whole process of agricultural production more intelligent.

Security: The physical carrier of agricultural energy internet is the integrated energy system, which coordinates the conversion of different forms of energy. When a certain form of energy is in short supply, other forms of energy are quickly transformed into this type of energy through relevant energy conversion technology, ensuring the safe and orderly progression of agricultural production.

The value of the energy internet includes the factors listed below.

Environmentally friendly. Agricultural production will produce a large amount of biomass, such as straw and manure, which are potentially useful and important production materials for the “source” of the agricultural energy internet. The use of straw gasification, biogas (straw and manure fermentation) power generation and other methods may reduce the dependence of the “source” on fossil energy.

Economic benefits. Due to the strong time-shiftability and unique flexibility of the agricultural load, the use of time-shiftability may reduce the investment in power facilities, maximize the use of power resources and achieve the economic operation of the power grid.

Currently, the high-pollution, high-energy consumption and low-efficiency agricultural production mode has been unable to easily meet the needs of economic and social development in China. Therefore, in the context of the energy internet, the development of the park-level agricultural energy internet is the key to achieving environment–energy–food cooperative security and is an important driving force to promote the transformation of agricultural production to a low-carbon, intelligence, intensive and efficient process. The emerging agricultural energy Internet in the park involves multiple disciplines, such as energy, agriculture and information and presents an interdisciplinary trend. In the future, the joint efforts of researchers in multiple disciplines are needed to further promote the development of the agricultural energy Internet in the park.

## 6. Conclusions

With the increasingly urgent cross integration of new energy development and modern agriculture, as well as the application of energy internet technology in agricultural and rural areas, the agricultural energy internet has become an extension of the development of integrated energy system and the inevitable trend to enhance the supply-side structural reform of rural energy. The conclusions of this work can be summarized as follows. (1) The integration of agriculture and new energy has replaced the traditional oil agriculture and become a new trend of agricultural energy development. New science and technology have intensified the integration of energy and agriculture. On the energy side, China has developed microbial fuel cells to replace fossil fuels. On the demand side, China has developed electric sterilization and fertilization to replace chemical sterilization and fertilization. (2) The integration of new energy and agriculture has become a new engineering mode with huge potential for economic development. Agricultural and forestry biomass power generation, agriculture–PV complementary, fishery–PV complementary are of great significance to improve agricultural economy and reduce carbon emissions. (3) The application of energy internet in agriculture can promote the realization of agricultural modernization, agricultural informatization, agricultural electrification and automation. Agricultural energy internet provides power for smart agriculture and becomes a framework for the development of agricultural energy systems in China.

## Figures and Tables

**Table 1:** Key technology of agricultural energy system.

Category	Technology Name	Core Principle	Benefit
New agricultural energy power generation technology	Agricultural textile power generation technology [124]	Conversion of kinetic energy into electric energy	a. Shading to protect crops b. Provide driving force for smart agriculture
	Concentrating photovoltaic and diffraction interferometry technology [125]	Diffraction and interference of light	a. Reduce water evaporation b. Improve power generation efficiency
	Microbial fuel cell technology [126]	Anaerobic respiration of microorganisms	a. Effectively degrade organic matter in biogas slurry b. Generate electric energy during degradation
Agricultural energy use technology	Greenhouse heating technology based on computational fluid dynamics (CFD) and the energy prediction model (EPM) [68]	CFD	a. Precisely control the environment of facility agriculture b. Reduce energy consumption
	Solar low temperature plasma nitrogen fixation technology [127]	Photovoltaic nitrogen fixation instead of chemical nitrogen fixation	a. Produce liquid nitrogen fertilizer b. No external energy required
	Plant nutrient solution sterilization technology based on the high-voltage pulsed electric field [128]	Physical sterilization instead of chemical sterilization	a. Promote crop growth b. Reduce energy consumption
	Technology for analyzing the safety of the agricultural micro-energy network [129]	Agrometeorology and energy meteorology	a. Provide safety warning for facility agricultural environment b. Provide safety warning for energy system
Internet of things (IoT) technology	Photovoltaic agricultural internet of things (PAIoT) technology [130]	Radio frequency identification (RFID), Wireless data communication	a. Monitor agricultural system status b. Monitor energy system status

## Data Availability

Not Applicable.

## References

[1] Cao X., Wu M., Guo X., Zheng Y., Gong Y., Wu N., Wang W. (2017). Assessing Water Scarcity in Agricultural Production System Based on the Generalized Water Resources and Water Footprint Framework. Sci. Total Environ..

[2] Zhang W., Cao Y., Zhu Y., Zheng J., Ji X., Xu Y., Wu Y., Hoitink A. (2018). Unravelling the Causes of Tidal Asymmetry in Deltas. J. Hydrol..

[3] Fei R., Lin B. (2016). Energy Efficiency and Production Technology Heterogeneity in China’s Agricultural Sector: A Meta-Frontier Approach. Technol. Forecast. Soc. Chang..

[4] Mostashari-Rad F., Nabavi-Pelesaraei A., Soheilifard F., Hosseini-Fashami F., Chau K.W. (2019). Energy Optimization and Green-House Gas Emissions Mitigation for Agricultural and Horticultural Systems in Northern Iran. Energy.

[5] Qureshi M.I., Awan U., Arshad Z., Rasli A.M., Zaman K., Khan F. (2016). Dynamic Linkages Among Energy Consumption, Air Pol-Lution, Greenhouse Gas Emissions and Agricultural Production in Pakistan: Sustainable Agriculture Key to Policy Success. Nat. Hazards.

[6] Jat H., Jat R., Nanwal R., Lohan S.K., Yadav A., Poonia T., Sharma P., Jat M. (2020). Energy Use Efficiency of Crop Residue Management for Sustainable Energy and Agriculture Conservation in NW India. Renew. Energy.

[7] Aydın B., Aktürk D. (2018). Energy Use Efficiency and Economic Analysis of Peach and Cherry Production Regarding Good Agricultural Practices in Turkey: A Case Study in Canakkale Province. Energy.

[8] Talukder B., Vanloon G.W., Hipel K.W. (2019). Energy Efficiency of Agricultural Systems in the Southwest Coastal Zone of Bangladesh. Ecol. Indic..

[9] Agathokleous E., Calabrese E.J. (2019). Hormesis Can Enhance Agricultural Sustainability in a Changing World. Glob. Food Secur..

[10] Liu C., Zhang Z., Liu S., Liu Q., Feng B., Tanzer J. (2019). Evaluating Agricultural Sustainability Based on the Water–Energy–Food Nexus in the Chenmengquan Irrigation District of China. Sustainability.

[11] Alam M., Murad W., Noman A.H.M., Ozturk I. (2016). Relationships Among Carbon Emissions, Economic Growth, Energy Consumption and Population Growth: Testing Environmental Kuznets Curve Hypothesis for Brazil, China, India and Indonesia. Ecol. Indic..

[12] Tjandra T.B., Ng R., Yeo Z., Song B. (2016). Framework and Methods to Quantify Carbon Footprint Based on an Office Environment in Singapore. J. Clean. Prod..

[13] Bekhet H.A., Othman N.S. (2017). Impact of Urbanization Growth on Malaysia CO_2_ Emissions: Evidence from the Dynamic Relationship. J. Clean. Prod..

[14] Reynolds T.W., Waddington S.R., Anderson C.L., Chew A., True Z., Cullen A.C. (2015). Environmental Impacts and Constraints Associated with the Production of Major Food Crops in Sub-Saharan Africa and South Asia. Food Secur..

[15] Lin B., Fei R. (2015). Analyzing Inter-Factor Substitution and Technical Progress in the Chinese Agricultural Sector. Eur. J. Agron..

[16] Liu Q., Liu B., Ambus P., Zhang Y., Hansen V., Lin Z., Shen D., Liu G., Bei Q., Zhu J. (2016). Carbon Footprint of Rice Production Under Biochar Amendment—A Case Study in a Chinese Rice Cropping System. GCB Bioenergy.

[17] Xue J., Huo Z., Huang Q., Wang F., Boll J., Huang G., Qu Z. (2018). Assessing Sustainability of Agricultural Water Saving in an Arid Area with Shallow Groundwater. Irrig. Drain..

[18] Cherni A., Jouini S.E. (2017). An ARDL approach to the CO2 Emissions, Renewable Energy and Economic Growth Nexus: Tunisian Evidence. Int. J. Hydrogen Energy.

[19] Rodriguez C.M., Rodas C.F., Munoz J.C., Casas A.F. (2019). A Multi-Criteria Approach for Comparison of Environmental Assessment Methods in the Analysis of the Energy Efficiency in Agricultural Production Systems. J. Clean. Prod..

[20] Abson D.J., Termansen M., Pascual U., Aslam U., Fezzi C., Bateman I. (2014). Valuing Climate Change Effects Upon UK Agricultural GHG Emissions: Spatial Analysis of a Regulating Ecosystem Service. Environ. Resour. Econ..

[21] Glenk K., Eory V., Colombo S., Barnes A. (2014). Adoption of Greenhouse Gas Mitigation in Agriculture: An Analysis of Dairy Farmers’ Perceptions and Adoption Behavior. Ecol. Econ..

[22] Pan H., Liu Y., Gao H. (2012). Impact of Agricultural Industrial Structure Adjustment on Energy Conservation and Income Growth in Western China: A Statistical Study. Ann. Oper. Res..

[23] Tieppo R.C., Romanelli T.L., Milan M., Sorensen C.A., Bochtis D. (2019). Modeling Cost and Energy Demand in Agricultural Machinery Fleets for Soybean and Maize Cultivated Using a No-Tillage System. Comput. Electron. Agric..

[24] National Bureau of Statistics (2018). China Statistical Yearbook.

[25] Kocaman A.S., Ozyoruk E., Taneja S., Modi V. (2020). A Stochastic Framework to Evaluate the Impact of Agricultural Load Flexibility on the Sizing of Renewable Energy Systems. Renew. Energy.

[26] Wu J., Ge Z., Han S., Xing L., Zhu M., Zhang J., Liu J. (2020). Impacts of Agricultural Industrial Agglomeration on China’s Agri-Cultural Energy Efficiency: A Spatial Econometrics Analysis. J. Clean. Prod..

[27] Chau J., Sowlati T., Sokhansanj S., Preto F., Melin S., Bi X. (2009). Economic Sensitivity of Wood Biomass Utilization for Green-House Heating Application. Appl. Energy.

[28] Qiao H., Zheng F., Jiang H., Dong K. (2019). The Greenhouse Effect of the Agriculture-Economic Growth-Renewable Energy Nexus: Evidence From g20 Countries. Sci. Total. Environ..

[29] Zorrilla-Munoz V., Petz M., Agullo-Tomas M.S. (2021). GARCH Model to Estimate the Impact of Agricultural Greenhouse Gas Emissions per Sociodemographic Factors and Cap in Spain. Environ. Dev. Sustain..

[30] Ridzuan N.H.A.M., Marwan N.F., Khalid N., Ali M.H., Tseng M.-L. (2020). Effects of Agriculture, Renewable Energy, and Economic Growth on Carbon Dioxide Emissions: Evidence of the Environmental Kuznets Curve. Resour. Conserv. Recycl..

[31] Zhang L., Pang J., Chen X., Lu Z.M. (2019). Carbon Emissions, Energy Consumption and Economic Growth: Evidence from the Agricultural Sector of China’s Main Grain-Producing Areas. Sci. Total Environ..

[32] Martinho V.J. (2016). Energy Consumption Across European Union Farms: Efficiency in Terms of Farming Output and Utilized Agricultural Area. Energy.

[33] Raeeni A.A., Hosseini S., Moghaddasi R. (2019). How Energy Consumption Is Related to Agricultural Growth and Export: An Econometric Analysis on Iranian Data. Energy Rep..

[34] Feng J., Feng L., Wang J., King C.W. (2020). Evaluation of the Onshore Wind Energy Potential in Mainland China—Based on GIS Modeling and EROI Analysis. Resour. Conserv. Recycl..

[35] Li J., Liu P., Li Z. (2020). Optimal Design and Techno-Economic Analysis of a Solar-Wind-Biomass off-Grid Hybrid Power System for Remote Rural Electrification: A Case Study of West China. Energy.

[36] Scarcioffolo A.R., Perobelli F.F., Chimeli A.B. (2018). Counterfactual Comparisons of Investment Options for Wind Power and Agri-Cultural Production in the United States: Lessons from Northern Ohio. Energy Econ..

[37] VanderWende B., Lundquist J.K. (2016). Could Crop Height Affect the Wind Resource at Agriculturally Productive Wind Farm Sites?. Bound. Layer Meteorol..

[38] Rehman S., Sahin A.Z. (2012). Wind-Solar PV Hybrid Power System with Battery Backup for Water Pumping in Remote Localities. Int. J. Green Energy.

[39] Mostafaeipour A., Rezaei M., Moftakharzadeh A., Qolipour M., Salimi M. (2019). Evaluation of Hydrogen Production by Wind en-Ergy for Agricultural and Industrial Sectors. Int. J. Hydrogen Energy.

[40] Mekhilef S., Faramarzi S.Z., Saidur R., Salam Z. (2013). The Application of Solar Technologies for Sustainable Development of Agricultural Sector. Renew. Sustain. Energy Rev..

[41] Tyagi V., Panwar N., Rahim N., Kothari R. (2012). Review on Solar Air Heating System with and Without Thermal Energy Storage System. Renew. Sustain. Energy Rev..

[42] Fabrizio E. (2012). Energy Reduction Measures in Agricultural Greenhouses Heating: Envelope, Systems and Solar Energy Collection. Energy Build.

[43] Chaysaz A., Seyedi S.R., Motevali A. (2019). Effects of Different Greenhouse Coverings on Energy Parameters of a Photovolta-IC–Thermal Solar System. Sol. Energy.

[44] Li C., Wang H., Miao H., Ye B. (2017). The Economic and Social Performance of Integrated Photovoltaic and Agricultural Green-Houses Systems: Case Study in China. Appl. Energy.

[45] Wu G., Yang Q., Zhang Y., Fang H., Feng C., Zheng H. (2020). Energy and Optical Analysis of Photovoltaic Thermal Integrated with Rotary Linear Curved Fresnel Lens Inside a Chinese Solar Greenhouse. Energy.

[46] Zhou Y., Chang F., Chang L., Lee W.D., Huang A., Xu C., Guo S. (2020). An Advanced Complementary Scheme of Floating Pho-Tovoltaic and Hydropower Generation Flourishing Water-Food-Energy Nexus Synergies. Appl. Energy.

[47] Feng C., Zheng H., Wang R. (2014). Development of Transparent Greenhouse Cover with Function of Generating Electricity by Surplus Light and Photovoltaic. Trans. Chin. Soc. Agric. Eng..

[48] Xue J. (2017). Photovoltaic Agriculture—New Opportunity for Photovoltaic Applications in China. Renew. Sustain. Energy Rev..

[49] Liu K., Wu P., Zhu D., Dai W., Li D., Cai S. (2017). Design and Test of Driving Power and Photovoltaic Power Matching for So-Lar-Driven Sprinkler Irrigation Unit. Trans. Chin. Soc. Agric. Eng..

[50] Li G., Jin Y., Akram M., Chen X. (2017). Research and Current Status of the Solar Photovoltaic Water Pumping System—A Review. Renew. Sustain. Energy Rev..

[51] He W., Zhang S., Hu Z., Zhang J., Liu X., Yu C., Yu H. (2020). Field Experimental Study on a Novel Beehive Integrated With Solar Thermal/Photovoltaic System. Sol. Energy.

[52] Anifantis A.S., Colantoni A., Pascuzzi S. (2017). Thermal Energy Assessment of a Small Scale Photovoltaic, Hydrogen and Geothermal Stand-Alone System for Greenhouse Heating. Renew. Energy.

[53] Cao K., Xu H., Zhang R., Xu D., Yan L., Sun Y., Xia L., Zhao J., Zou Z., Bao E. (2019). Renewable and Sustainable Strategies for Improving the Thermal Environment of Chinese Solar Greenhouses. Energy Build..

[54] Ferrari G., Pezzuolo A., Nizami A.-S., Marinello F. (2020). Bibliometric Analysis of Trends in Biomass for Bioenergy Research. Energies.

[55] Amaducci S., Yin X., Colauzzi M. (2018). Agrivoltaic Systems to Optimise Land Use for Electric Energy Production. Appl. Energy.

[56] Wang W., Ouyang W., Hao F., Liu G. (2017). Temporal-Spatial Variation Analysis of Agricultural Biomass and Its Policy Implication as an Alternative Energy in Northeastern China. Energy Policy.

[57] Liu D., Liu M., Xiao B., Guo X., Niu D., Qin G., Jia H. (2020). Exploring Biomass Power Generation’s Development Under en-Couraged Policies in China. J. Clean. Prod..

[58] Guan Y., Tai L., Cheng Z., Chen G., Yan B., Hou L. (2020). Biomass Molded Fuel in China: Current Status, Policies and Suggestions. Sci. Total. Environ..

[59] Sun J., Chen J., Xi Y., Hou J. (2011). Mapping the Cost Risk of Agricultural Residue Supply for Energy Application in Rural China. J. Clean. Prod..

[60] Wang C., Zhang L., Zhou P., Chang Y., Zhou D., Pang M., Yin H. (2019). Assessing the Environmental Externalities for Biomass-and Coal-Fired Electricity Generation in China: A Supply Chain Perspective. J. Environ. Manag..

[61] Wei J., Liang G., Alex J., Zhang T., Ma C. (2020). Research Progress of Energy Utilization of Agricultural Waste in China: Bibliometric Analysis by Citespace. Sustainability.

[62] Dai X., Hua Y., Liu R., Chen S., Li H., Dai L., Cai C. (2020). Biomethane Production by Typical Straw Anaerobic Digestion: Deep Insights of Material Compositions and Surface Properties. Bioresour. Technol..

[63] Cheng H.-H., Whang L.-M., Chung M.-C., Chan K.-C. (2016). Biological Hydrogen and Methane Production from Bagasse Bioethanol Fermentation Residues Using a Two-Stage Bioprocess. Bioresour. Technol..

[64] Dong L., Cao G., Zhao L., Liu B., Ren N. (2018). Alkali/Urea Pretreatment of Rice Straw at Low Temperature for Enhanced Biological Hydrogen Production. Bioresour. Technol..

[65] Jankowski K.J., Sokólski M.M., Dubis B., Załuski D., Szempliński W. (2020). Sweet Sorghum—Biomass Production and Energy Balance at Different Levels of Agricultural Inputs. a Six-Year Field Experiment in North-Eastern Poland. Eur. J. Agron..

[66] Forster-Carneiro T., Berni M.D., Lachos-Perez D., Dorileo I.L., Rostagno M.A. (2017). Characterization and Analysis of Specific Energy Consumption in the Brazilian Agricultural Sector. Int. J. Environ. Sci. Technol..

[67] Ghobadpour A., Boulon L., Mousazadeh H., Malvajerdi A.S., Rafiee S. (2019). State of the Art of Autonomous Agricultural off-Road Vehicles Driven by Renewable Energy Systems. Energy Procedia.

[68] Chen J., Xu F., Tan D., Shen Z., Zhang L., Ai Q. (2015). A Control Method for Agricultural Greenhouses Heating Based on Compu-Tational Fluid Dynamics and Energy Prediction Model. Appl. Energy.

[69] Vadiee A., Martin V. (2013). Energy Analysis and Thermoeconomic Assessment of the Closed Greenhouse—The Largest Commercial Solar Building. Appl. Energy.

[70] Bot G.P. (2001). Developments in Indoor Sustainable Plant Production with Emphasis on Energy Saving. Comput. Electron. Agric..

[71] Sethi V., Sharma S. (2008). Survey and Evaluation of Heating Technologies for Worldwide Agricultural Greenhouse Applications. Sol. Energy.

[72] Sandgani M.R., Sirouspour S. (2018). Priority-Based Microgrid Energy Management in a Network Environment. IEEE Trans. Sustain. Energy.

[73] Abrishambaf O., Faria P., Gomes L., Vale Z. (2020). Agricultural Irrigation Scheduling for a Crop Management System Considering Water and Energy Use Optimization. Energy Rep..

[74] Malik W., Dechmi F. (2019). DSSAT Modelling for Best Irrigation Management Practices Assessment Under Mediterranean Conditions. Agric. Water Manag..

[75] Barak S., Yousefi M., Maghsoudlou H., Jahangiri S. (2015). Energy and GHG Emissions Management of Agricultural Systems Using Multi Objective Particle Swarm Optimization Algorithm: A Case Study. Stoch. Environ. Res. Risk Assess..

[76] Ojha T., Misra S., Raghuwanshi N.S. (2015). Wireless Sensor Networks for Agriculture. Comput. Electron. Agric..

[77] Gutierrez J., Villa-Medina J.F., Nieto-Garibay A., Porta-Gandara M.A. (2013). Automated Irrigation System Using a Wireless Sensor Network and GPRS Module. IEEE Trans. Instrum. Meas..

[78] Munir M.S., Bajwa I.S., Naeem M.A., Ramzan B. (2018). Design and Implementation of an IoT System for Smart Energy Consumption and Smart Irrigation in Tunnel Farming. Energies.

[79] Jackson T.M., Khan S., Hafeez M. (2010). A Comparative Analysis of Water Application and Energy Consumption at the Irrigated Field Level. Agric. Water Manag..

[80] Caglar A.E. (2020). The Importance of Renewable Energy Consumption and FDI Inflows in Reducing Environmental Degradation: Bootstrap ARDL Bound Test in Selected 9 Countries. J. Clean. Prod..

[81] Dogan E., Inglesi-Lotz R. (2017). Analyzing the Effects of Real Income and Biomass Energy Consumption on Carbon Dioxide (CO_2_) Emissions: Empirical Evidence from the Panel of Biomass-Consuming Countries. Energy.

[82] Du K., Lin B. (2015). Understanding the Rapid Growth of China’s Energy Consumption: A Comprehensive Decomposition Framework. Energy.

[83] Zhen W., Qin Q., Wei Y. (2017). Spatio-Temporal Patterns of Energy Consumption-Related GHG Emissions in China’s Crop Production Systems. Energy Policy.

[84] Zhang J., Liu Y., Chang Y., Zhang L. (2017). Industrial Eco-Efficiency in China: A Provincial Quantification Using Three-Stage Data Envelopment Analysis. J. Clean. Prod..

[85] Zheng J., Wang W., Chen D., Cao X., Xing W., Ding Y., Dong Q., Zhou T. (2018). Exploring the Water–Energy–Food Nexus from a Perspective of Agricultural Production Efficiency Using a Three-Stage Data Envelopment Analysis Modelling Evaluation Method: A Case Study of the Middle and Lower Reaches of the Yangtze River, China. Hydrol. Res..

[86] Duan C., Wang X., Shu S., Jing C., Chang H. (2014). Thermodynamic Design of Stirling Engine Using Multi-Objective Particle Swarm Optimization Algorithm. Energy Convers. Manag..

[87] Wang Y., Niu H., Yang L., Wang W., Liu F. (2018). An Optimization Method for Local Consumption of Photovoltaic Power in a Facility Agriculture Micro Energy Network. Energies.

[88] Cui Z., Zhang F., Chen X., Dou Z., Li J. (2010). In-Season Nitrogen Management Strategy for Winter Wheat: Maximizing Yields, Minimizing Environmental Impact in an Over-Fertilization Context. Field Crop. Res..

[89] Zhang B.Z., Kang S.Z., Zhang L., Du T.S., Li S.E., Yang X.Y. (2007). Estimation of Seasonal Crop Water Consumption in a Vineyard Using Bowen Ratio-Energy Balance Method. Hydrol. Process..

[90] Xiao K., Xiao D., Luo X. (2010). Smart Water-Saving Irrigation System in Precision Agriculture Based on Wireless Sensor Network. Trans. Chin. Soc. Agric. Eng..

[91] Fei R., Lin B. (2017). Estimates of Energy Demand and Energy Saving Potential in China’s Agricultural Sector. Energy.

[92] Shine P., Scully T., Upton J., Murphy M. (2018). Multiple Linear Regression Modelling of On-Farm Direct Water and Electricity Consumption on Pasture Based Dairy Farms. Comput. Electron. Agric..

[93] Todde G., Murgia L., Caria M., Pazzona A. (2017). Dairy Energy Prediction (DEP) Model: A Tool for Predicting Energy Use and Related Emissions and Costs in Dairy Farms. Comput. Electron. Agric..

[94] Shine P., Scully T., Upton J., Murphy M. (2019). Annual Electricity Consumption Prediction and Future Expansion Analysis on Dairy Farms Using a Support Vector Machine. Appl. Energy.

[95] Hosseinzadeh-Bandbafha H., Nabavi-Pelesaraei A., Shamshirband S. (2017). Investigations of Energy Consumption and Greenhouse Gas Emissions of Fattening Farms Using Artificial Intelligence Methods. Environ. Prog. Sustain. Energy.

[96] Sefeedpari P., Rafiee S., Akram A., Komleh S.H. (2014). Modeling Output Energy Based on Fossil Fuels and Electricity Energy Con-Sumption on Dairy Farms of Iran. Comput. Electron. Agric..

[97] O’Brien D., Shalloo L., Patton J., Buckley F., Grainger C., Wallace M. (2012). A Life Cycle Assessment of Seasonal Grass-Based and Confinement Dairy Farms. Agric. Syst..

[98] Ilyas H.M.A., Safa M., Bailey A., Rauf S., Pangborn M. (2019). The Carbon Footprint of Energy Consumption in Pastoral and Barn Dairy Farming Systems: A Case Study from Canterbury, New Zealand. Sustainability.

[99] Alberti L., Antelmi M., Angelotti A., Formentin G. (2018). Geothermal Heat Pumps for Sustainable Farm Climatization and Field Irrigation. Agric. Water Manag..

[100] Islam M., Mun H.-S., Bostami A.B.M.R., Ahmed S.T., Park K.-J., Yang C.-J. (2016). Evaluation of a Ground Source Geothermal Heat Pump to Save Energy and Reduce CO_2_ and Noxious Gas Emissions in a Pig House. Energy Build..

[101] Xie Q., Ni J.-Q., Bao J., Su Z. (2019). A Thermal Environmental Model for Indoor Air Temperature Prediction and Energy Consumption in Pig Building. Build. Environ..

[102] Yang Q., Wu X., Yang H., Zhang S., Chen H. (2012). Nonrenewable Energy Cost and Greenhouse Gas Emissions of a “Pig-Biogas-Fish” System in China. Sci. World J..

[103] Du L., Hu C., Yang C., Yang L., Du H., Li Q., Yu C., Xie L., Jiang X. (2020). Investigation of a Preliminary Ventilation Ener-GY-Recovery System for Poultry Houses. Comput. Electron. Agric..

[104] Du L., Yang C., Dominy R., Yang L., Hu C., Du H., Li Q., Yu C., Xie L., Jiang X. (2019). Computational Fluid Dynamics Aided Investigation and Optimization of a Tunnel-Ventilated Poultry House in China. Comput. Electron. Agric..

[105] Wang K., Pantaleo A.M., Herrando M., Faccia M., Pesmazoglou I., Franchetti B.M., Markides C.N. (2020). Spectral-Splitting Hybrid PV-Thermal (PVT) Systems for Combined Heat and Power Provision to Dairy Farms. Renew. Energy.

[106] Bastardie F., Nielsen J.R., Andersen B.S., Eigaard O.R. (2010). Effects of Fishing Effort Allocation Scenarios on Energy Efficiency and Profitability: An Individual-Based Model Applied to Danish Fisheries. Fish. Res..

[107] Ziegler F., Hansson P.-A. (2003). Emissions from Fuel Combustion in Swedish Cod Fishery. J. Clean. Prod..

[108] Basurko O.C., Gabiña G., Uriondo Z. (2013). Energy Performance of Fishing Vessels and Potential Savings. J. Clean. Prod..

[109] Grimaldo E., Pedersen R., Sistiaga M. (2015). Energy Consumption of Three Different Trawl Configurations Used in the Barents Sea Demersal Trawl Fishery. Fish. Res..

[110] Schau E.M., Ellingsen H., Endal A., Aanondsen S.A. (2009). Energy Consumption in the Norwegian Fisheries. J. Clean. Prod..

[111] Alzahrani A., Petri I., Rezgui Y., Ghoroghi A. (2020). Developing Smart Energy Communities Around Fishery Ports: Toward Zero-Carbon Fishery Ports. Energies.

[112] Ramos V., Carballo R., Álvarez M., Sánchez M., Iglesias G. (2014). A Port Towards Energy Self-Sufficiency Using Tidal Stream Power. Energy.

[113] Misra A., Panchabikesan K., Gowrishankar S.K., Ayyasamy E., Ramalingam V. (2017). GHG Emission Accounting and Mitigation Strategies to Reduce the Carbon Footprint in Conventional Port Activities—A Case of the Port of Chennai. Carbon Manag..

[114] Reynolds J., Rezgui Y., Hippolyte J.-L. (2017). Upscaling Energy Control from Building to Districts: Current Limitations and Future Perspectives. Sustain. Cities Soc..

[115] Reynolds J., Rezgui Y., Kwan A., Piriou S. (2018). A Zone-Level, Building Energy Optimisation Combining an Artificial Neural Network, a Genetic Algorithm, and Model Predictive Control. Energy.

[116] Love D.C., Fry J.P., Li X., Hill E.S., Genello L., Semmens K.J., Thompson R.E. (2015). Commercial Aquaponics Production and Profitability: Findings from an International Survey. Aquaculture.

[117] Al-Hafedh Y.S., Alam A., Beltagi M.S. (2008). Food Production and Water Conservation in a Recirculating Aquaponic System in Saudi Arabia at Different Ratios of Fish Feed to Plants. J. World Aquac. Soc..

[118] Le A.T., Wang Y., Wang L., Ta V.C., Li D. (2020). Numerical Investigation on a Low Energy-Consumption Heating Method for Recirculating Aquaponic Systems. Comput. Electron. Agric..

[119] Delaide B., Delhaye G., Dermience M., Gott J., Soyeurt H., Jijakli M.H. (2017). Plant and Fish Production Performance, Nutrient Mass Balances, Energy and Water Use of the PAFF Box, a Small-Scale Aquaponic System. Aquac. Eng..

[120] Xing Y., Lin J. (2011). Application of Electrochemical Treatment for the Effluent from Marine Recirculating Aquaculture Systems. Procedia Environ. Sci..

[121] Sun J., Li N., Yang P., Zhang Y., Yuan Y., Lu X., Zhang H. (2020). Simultaneous Antibiotic Degradation, Nitrogen Removal and Power Generation in a Microalgae-Bacteria Powered Biofuel Cell Designed for Aquaculture Wastewater Treatment and Energy Recovery. Int. J. Hydrogen Energy.

[122] Yuan H., Zhou P., Mei N. (2015). Performance Analysis of a Solar-Assisted OTEC Cycle for Power Generation and Fishery Cold Storage Refrigeration. Appl. Therm. Eng..

[123] Jiang Q., Jie Y., Han Y., Gao C., Zhu H., Willander M., Zhang X., Cao X. (2015). Self-Powered Electrochemical Water Treatment System for Sterilization and Algae Removal Using Water Wave Energy. Nano Energy.

[124] Jiang C., Li X., Ying Y., Ping J. (2020). A Multifunctional TENG Yarn Integrated into Agrotextile for Building Intelligent Agriculture. Nano Energy.

[125] Liu W., Liu L., Guan C., Zhang F., Li M., Lv H., Yao P., Ingenhoff J. (2018). A Novel Agricultural Photovoltaic System Based on Solar Spectrum Separation. Sol. Energy.

[126] Wang F., Zhang D., Shen X., Liu W., Yi W., Li Z., Liu S. (2019). Synchronously Electricity Generation and Degradation of Biogas Slurry Using Microbial Fuel Cell. Renew. Energy.

[127] Yang J., Li T., Zhong C., Guan X., Hu C. (2016). Nitrogen Fixation in Water Using Air Phase Gliding Arc Plasma. J. Electrochem. Soc..

[128] Zhong C., Guan X., Fan Z., Song W., Chen R., Wang Y., Sun X., He S. (2019). Pulsed Electric Field Disinfection Treatment of Fusarium Oxysporum in Nutrient Solution. Water Supply.

[129] Fu X., Yang D., Guo Q., Sun H. (2020). Security Analysis of a Park-Level Agricultural Energy Network Considering Agrometeorology and Energy Meteorology. CSEE J. Power Energy Syst..

[130] Huang K., Shu L., Li K., Yang F., Han G., Wang X., Pearson S. (2020). Photovoltaic Agricultural Internet of Things Towards Realizing the Next Generation of Smart Farming. IEEE Access.

[131] Wang J. (2019). Study on the Photovoltaic Agriculture Engineering Mode and Its Application in China. Ph.D. Thesis.

[132] Kubuqi Desert Ecological Solar Energy Control Sofa Power Comprehensive Demonstration Project Phase III Project 200MWp Photovoltaic Desert Control Project Grid-connected Power Generation. http://www.kubuqiforum.org/index.php?menu=11&id=205.

[133] The “Blue Ocean” in the Desert Feels Chint Kubuqi Desert Photovoltaic Power Station. https://www.ne21.com/news/show-117474.html.

[134] Huawei Smart Photovoltaic Helps Build Huzhou’s Largest Complementary Photovoltaic Power Station. http://www.chinaden.cn/news_nr.asp?id=5549&Small_Class=7.

[135] Annual Power Generation Exceeds 200 Million kWh, Zhejiang Huzhou “Fishing and Light Complementation” Creates Ecological Beauty. http://guangfu.bjx.com.cn/news/20180619/906656.shtml.

[136] The World’s Largest Agricultural-Photovoltaic Complementary Photovoltaic Power Station Was Completed and Put into Operation in China. http://www.zc-sensor.com/news/new_3143.

[137] Ningxia Baofeng: 1GW Agricultural and Photovoltaic Complementary Photovoltaic Power Station Successfully Connected to the Grid for Power Generation with an Average Annual Income of 360 Million Yuan. https://news.solarbe.com/201706/07/114246.html.

[138] Huadian Biomass Power Generation Project: Turning Waste into Treasure, Green Cycle. http://jxj.xiangyang.gov.cn/zxzx/gzdt/202007/t20200708_2197000.shtml.

[139] The Country’s First Coal-Fired Coupling Agricultural and Forestry Biomass Power Generation Technical Reform Pilot Project Suc-Cessfully Passed the Evaluation and Inspection. http://www.chd.com.cn/webfront/webpage/web/contentPage/id/88a16c443515407c823963cd7a666142.

[140] Fu X., Zhou Y., Sun H., Wang Y. (2020). Park-Level Agricultural Energy Internet: Concept, Characteristic and Application Value. Trans. Chin. Soc. Agric. Eng..

[141] Fu X., Zhou Y., Sun H., Guo Q. (2020). Online Security Analysis of a Park-Level Agricultural Energy Internet: Review and Prospect. Proc. Chin. Soc. Electr. Eng..

